# Quality Assurance Investigations and Impurity Characterization during Upscaling of [^177^Lu]Lu-PSMA^I&T^

**DOI:** 10.3390/molecules28237696

**Published:** 2023-11-21

**Authors:** Stefan Schmitl, Julia Raitanen, Stephan Witoszynskyj, Eva-Maria Patronas, Lukas Nics, Marius Ozenil, Victoria Weissenböck, Thomas L. Mindt, Marcus Hacker, Wolfgang Wadsak, Marie R. Brandt, Markus Mitterhauser

**Affiliations:** 1 Department of Biomedical Imaging and Image-Guided Therapy, Division of Nuclear Medicine, Medical University of Vienna, 1090 Vienna, Austria; 2 Ludwig Boltzmann Institute Applied Diagnostics, AKH Wien c/o Sekretariat Nuklearmedizin, 1090 Vienna, Austria; 3 Department of Inorganic Chemistry, Faculty of Chemistry, University of Vienna, 1090 Vienna, Austria; 4 Vienna Doctoral School of Chemistry (DoSChem), University of Vienna, 1090 Vienna, Austria; 5 Joint Applied Medicinal Radiochemistry Facility, University of Vienna & Medical University of Vienna, 1090 Vienna, Austria

**Keywords:** [^177^Lu]Lu-PSMA^-I&T^, radiolysis, radioligand therapy, upscaling, quality assurance, HPLC, UPLC-MS

## Abstract

[^177^Lu]Lu-PSMA^I&T^ is widely used for the radioligand therapy of metastatic castration-resistant prostate cancer (mCRPC). Since this kind of therapy has gained a large momentum in recent years, an upscaled production process yielding multiple patient doses in one batch has been developed. During upscaling, the established production method as well as the HPLC quality control were challenged. A major finding was a correlation between the specific activity and the formation of a pre-peak, presumably caused by radiolysis. Hence, nonradioactive reference standards were irradiated with an X-ray source and the formed pre-peak was subsequently identified as a deiodination product by UPLC-MS. To confirm the occurrence of the same deiodinated side product in the routine batch, a customized deiodinated precursor was radiolabeled and analyzed with the same HPLC setup, revealing an identical retention time to the pre-peak in the formerly synthesized routine batches. Additionally, further cyclization products of [^177^Lu]Lu-PSMA^I&T^ were identified as major contributors to radiochemical impurities. The comparison of two HPLC methods showed the likelihood of the overestimation of the radiochemical purity during the synthesis of [^177^Lu]Lu-PSMA^I&T^. Finally, a prospective cost reduction through an optimization of the production process was shown.

## 1. Introduction

[^177^Lu]Lu-PSMA^I&T^ (INN: Lutetium (^177^Lu) zadavotide guraxetan) [1] is one of the main PSMA-directed radiopharmaceuticals that are successfully used for the treatment of metastatic castration-resistant prostate cancer in nuclear medicine departments around the world [2]. Lutetium-177 decays via β^−^/γ emission to its daughter nuclide hafnium-176 (^176^Hf). With a physical half-life of 6.647 days and a maximum tissue penetration range of <2 mm of its emitted β particles, it displays beneficial properties for the deposition of high radiation doses to tumor lesions and metastases while sparing the surrounding tissue [3].

The success of PSMA-directed radioligand therapy is not only reflected by the very recent approval of [^177^Lu]Lu-PSMA-617 (Pluvicto^®^) for mCRPC in the US and EU, but also by the increasing clinical demand for comparable agents [2,4,5]. In the literature, administered activities ranging from 3.7 to 9.3 GBq were described for [^177^Lu]Lu-PSMA therapy in metastatic castration-resistant prostate cancer (mCRPC) [6]. In our clinic, 7.4 GBq of [^177^Lu]Lu-PSMA^I&T^ every six weeks represents the current standard in mCRPC treatment, based on the marketing authorization of Pluvicto^®^. However, lower levels have been administered in earlier stages of prostate carcinoma in recent pilot studies [7,8].

In the Division of Nuclear Medicine at the General Hospital of Vienna, the number of mCRPC patients referred to [^177^Lu]Lu-PSMA^I&T^ therapy under named patient use has constantly increased throughout recent years. This development is in accordance with a recent study that showed an increasing prevalence of mCRPC in the United States, with stable incidence rates between 2010 and 2017, mostly attributable to the introduction of novel therapies (e.g., taxane-based chemotherapy, androgen deprivation therapy) [9]. To ensure the supplying of all our patients with this treatment using the available resources, it became necessary to upscale our production process from two patients per batch to at least four patients per batch (18 to 36 GBq starting activity/batch). Due to the increasing amount of radioactivity, radiolysis was suspected to constitute a major problem with regard to radiochemical purity results during upscaling.

**Figure 1:**
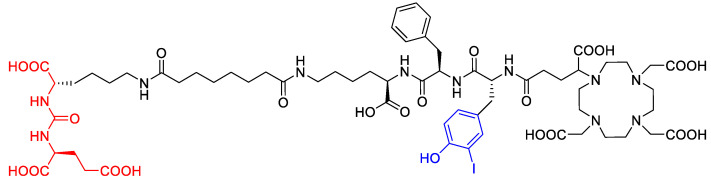
Molecular structure of PSMA^I&T^ with Glu-urea-Lys-binding motif (red, left) and iodotyrosine moiety (center, blue).

The degradation of a radiopharmaceutical under the influence of its incorporated radionuclide (radiolysis) is a phenomenon known to occur during the production of [^177^Lu]Lu-PSMA^I&T^ and other radiopharmaceuticals labeled with α or β^−^ emitters. The process is mediated predominantly by solvated electrons, radicals and highly reactive molecules that result from the irradiation of water and thereby formed reactive oxygen species (ROS). To a lower extent, radiolysis is mediated by direct effects of radiation on the radiopharmaceutical. Different radiolysis quenchers have been investigated to maintain the radiochemical purity of therapeutic radiopharmaceuticals during storage [10]. The major radiolysis product of [^177^Lu]Lu-PSMA^I&T^ was recently suggested to be the result of deiodination of the iodotyrosin moiety (see Figure 1) [11].

For [^177^Lu]Lu-PSMA^I&T^, the radiolabeling with up to 30 GBq in the presence of gentisic acid, ascorbic acid and sodium acetate followed by the dilution of the reaction mixture with an ascorbic acid solution showed excellent radiochemical purity results of >97% after storage for 30 h [12]. The effectiveness of ascorbic acid/gentisic acid combination buffers can be explained by the oxidation of gentisic acid via ROS and the subsequent reduction of the primary radicals via ascorbic acid, hence sparing the radiopetide [13].

Apart from radiolysis, the chemical rearrangement of the product was also considered to have a possible influence on the formation of radioactive side products, although not necessarily related to the increased radioactivity amounts. Recently, the heat-dependent formation of hydantoins via cyclization of the PSMA binding motif was shown to occur during the radiosynthesis of [^177^Lu]Lu-PSMA-617. These hydantoins showed no binding affinity to PSMA and were rapidly excreted by the kidneys. It was shown that these byproducts constitute >2% of the sum of radiochemical impurities, even under optimized conditions [14]. To the best of our knowledge, the formation of hydantoins in the PSMA binding motif has not been described for [^177^Lu]Lu-PSMA^I&T^ yet. Given the likelihood of the formation of these by-products in [^177^Lu]Lu-PSMA-I&T, we suspect that an overestimation of radiochemical purity could be prevalent throughout the existing literature, possibly due to the use of unsuitable HPLC methods for the detection of the cyclization products.

The aim of this study was therefore to investigate the formation and identity of radiolysis and cyclization products during the upscaling of [^177^Lu]Lu-PSMA^I&T^ production.

## 2. Results and Discussion

### 2.1. Investigation of the Production Process of [^177^Lu]Lu-PSMA^I&T^

**Figure 2:**
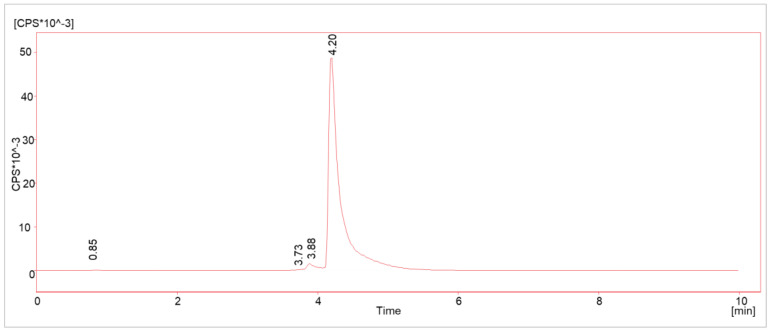
Typical γ-HPLC chromatogram (method 1) of [^177^Lu]Lu-PSMA^I&T^ with main product (R_t_ = 4.20 min) and radiolysis-induced side product (R_t_ = 3.88 min).

Due to the recent publications on radiolysis-generated side products [10,12,15] in Lutetium-177-labeled radiopharmaceuticals, the radiochemical purity (RCP) depending on the radioactivity concentration was defined as a key parameter to be investigated and prioritized in the upscaling process. Hence, several batches with different starting activity concentrations (AC, 0.5–2.5 GBq/mL) were analyzed with HPLC method 1 (see chapter “Methods”) for possible radiolysis products. The batches were produced according to the method referred to as “original method” (see Section 3.4). A pre-peak (R_t_ = 3.88 min) was detected in all cases and all activity concentrations, with a slightly shorter retention time than the main product (R_t_ = 4.20 min; see Figure 2). Its integral was below the in-house limit of 5% by that time for ACs up to 1 GBq/mL. No other peaks were detected in the radioactivity channel.

**Figure 3:**
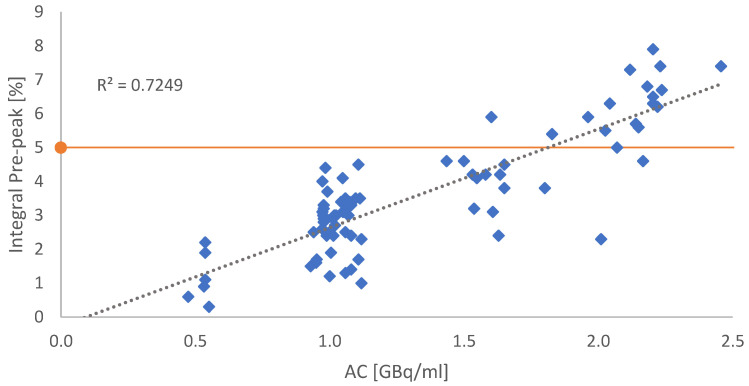
Graphical correlation between the integral of the suspected radiolysis-induced side product and the AC. Each blue dot refers to a single production batch. The orange line indicates the in-house specification benchmark of 5% impurities. All dots above the red line indicate batches with RCP < 95%.

Depending on the AC, the integral of the impurity was rising, revealing a correlation between the pre-peak and the AC (see Figure 3).

### 2.2. HPLC Optimization Studies and HPLC Validation

**Figure 4:**
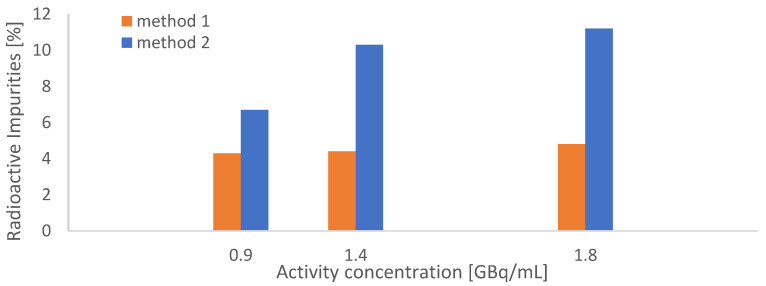
Comparison between HPLC methods 1 and 2 through simultaneous measurements of [^177^Lu]Lu-PSMA^I&T^ batches; three different batches with increasing activities (18–36 GBq, AC = 1–2 GBq/mL, N = 1) were synthesized according to the original synthetic procedure.

In the next step, our HPLC quality control methods were revised, as the relatively poor separation of the product from the radiolysis pre-peak was identified as problematic. Hence, the HPLC method described as “method C” by Martin et al. [14] was slightly modified and the resulting method will be called “method 2” throughout this text [14] (for details see the chapter “Methods”). The method was originally used to detect cyclization products of [^177^Lu]Lu-PSMA-617 in patient urine samples. For comparison, simultaneous measurements of test batches in two identical HPLC setups were conducted to compare the performance of method 1 and method 2. The direct comparison showed significant underestimations of the presence of impurities by method 1 (see Figure 4).

The validation results of HPLC method 2 are displayed in Table 1.

To improve the radiochemical purity of the product, the need for a modified production process and storage formulation to suppress the suspected radiolysis became obvious. As described in the section “Methods: Radiosyntheses”, a manual process described by Di Orio et al. [12] was modified and automatized (called “adapted method” in this text, whereas “original method” describes our previously used procedure, for details see Section 3.4).

**Figure 5:**
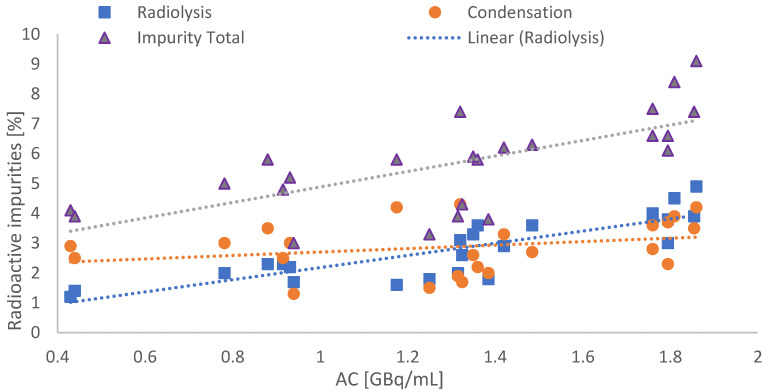
Total amount of radioactive impurities by using the new production and HPLC method in dependence of AC (blue dots: radiolysis pre-peak; orange circle: condensation side products; purple triangles: total impurities).

The implementation of the new production method combined with HPLC method 2 showed a maximum of ≤10% total impurity for syntheses with a maximum AC of 1.8 GBq/mL in the product solution, as displayed in Figure 5.

**Figure 6:**
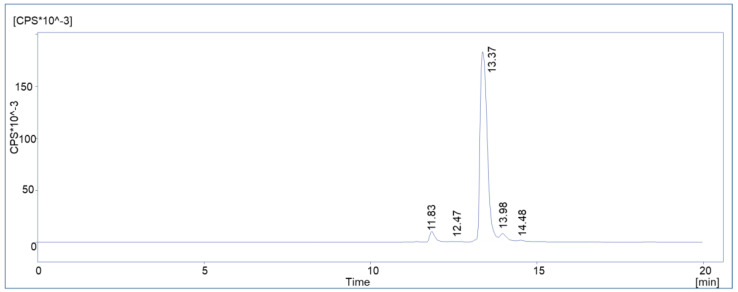
Representative chromatogram of a routine batch of [^177^Lu]Lu-PSMA^I&T^ with HPLC method 2.

It is also shown that, in addition to the baseline separated radiolysis product, hydrophobic substances eluting after the main product additionally appeared in HPLC method 2 (contribution of 2.87 ± 0.85% to total impurities) and furthermore formed independently from the activity concentration. A typical HPLC chromatogram acquired with method 2 is displayed in Figure 6.

Stability measurements were performed on three batches containing 8.6, 26.3 and 26.5 GBq. All batches showed >95% radiochemical purity (HPLC) after 4 h. Long term stability studies were not conducted since no shipment to other facilities or commercial use was intended.

In the next step, the radiochemical by-products were identified.

### 2.3. Identification of the Radiolysis By-Products: Irradiation Experiments and Coelution

**Figure 7:**
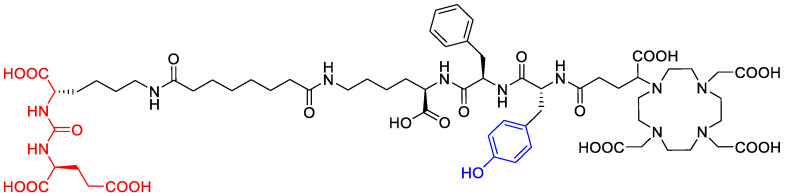
Molecular structure of the deiodinated PSMA^I&T^ by-product with the intact binding motif and missing iodine atom on the tyrosine side chain.

At this stage of the project, the identity of the pre-peak in [^177^Lu]Lu-PSMA^I&T^ was not yet clear (a study by Kraihammer et al. with a similar experimental setup has been published since then [11,13]). Due to the almost linear correlation between the intensity of the by-product and the radioactivity concentrations as described above, radiolysis was highly likely to be the cause of the formation. The dissociation of the iodine atom in the iodotyrosine moiety of the molecule (see Figure 7), which was shown to be caused by reactive oxygen species (ROS) induced by external radiation in aqueous media [16], was suspected to be the most probable cause.

Hence, radiolysis was investigated by simulation. The intact Gd-PSMA^I&T^ was at first subjected to LC-MS and showed a retention time of 7.721 min and [M+2H]^2+^ was found to be 828.30 (calculated: 828.31).

Then, the non-radioactive reference compound Gd-PSMA^I&T^ was irradiated in both the reaction buffer (ascorbate buffer from POLATOM) as well as in the reaction buffer plus ethanol with our in-house X-ray irradiation device (see “methods: Radiosyntheses”). Due to the similar chemical behavior and lower cost, a gadolinium salt was used as a surrogate for lutetium.

**Figure 8:**
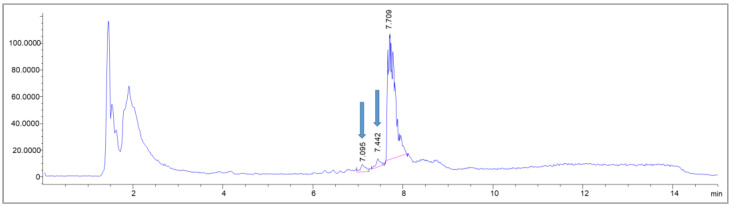
MS trace of an exemplary LC-MS measurement of a sample without buffer adjustments, irradiated with X-rays equivalent to 130 Gy. The blue arrows indicate the pre-peaks newly formed due to the irradiation.

First, a simplified dose calculation was performed under the following four assumptions: 1. The β^−^ emitter Lutetium-177 was causing substance damage comparable to X-rays. 2. The radionuclide was evenly distributed throughout the reaction vessel in 19.6 mL total volume. 3. Only ß^−^ particles contributed to the total dose. 4. The storage time was 45 min. The physical dose for the ACs of 0.4, 0.9, 1.8 and 2 GBq/mL, corresponding to batch activities of 7, 18, 36 and 40 GBq per batch (for the calculation, see the chapter “Dose Calculations” in the Section 3), was calculated. Then, the samples were irradiated with the calculated doses and analyzed via LC-MS to identify potentially formed side products. In the samples without ethanol, two side products could be detected, whose intensities increased with rising dose equivalents (Figure 8) and were barely present in the ethanol containing samples kept for the same time in the same buffer.

**Figure 9:**
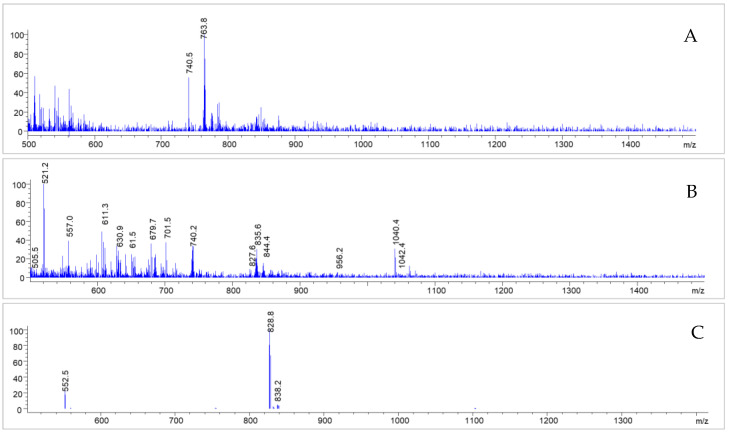
MS spectra corresponding to the impurities detected (R_t_ = 7.095 (**A**); 7.442 (**B**) and 7.709 (**C**)); after irradiation of Gd-PSMA^I&T^ without adjusted buffer system, as described further above.

In the corresponding MS spectra, one of the pre-peaks (R_t_ = 7.095 min) could in fact be identified as the deiodinated Gd-PSMA^I&T^ (see Figure 9) with a molecular mass of 1527 (*m*/*z* = 763.8 [M-I+H]^+2^). The other side product, with a retention time of R_t_ = 7.442 min, showed multiple mass peaks (*m*/*z* = 104.4; 740.8; 521.2) and could not be identified unambiguously (Figure 9). The main peak, however, showed intact Gd-PSMA^I&T^ (R_t_ = 7.709 min; *m*/*z* = 828.8 [M+2H]^2+^; 839.1 [M+H+Na]^2+^; see Figure 8 and Figure 9).

**Figure 10:**
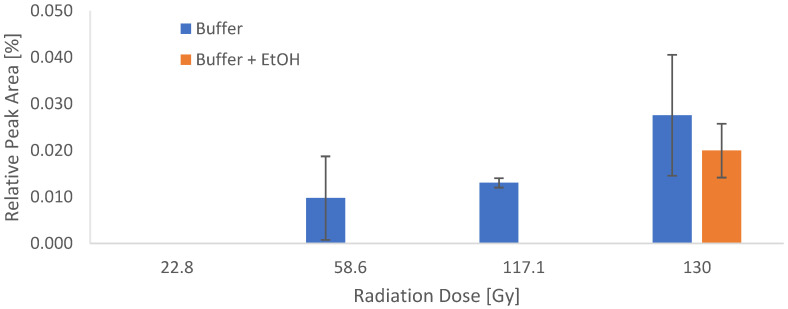
Integrals of the deiodinated compound determined via UPLC-MS in correlation with the applied dose in the two different reaction buffers. N = 1, performed in duplicate.

To quantify the effect of the irradiation on Gd-PSMA^I&T^ in the two different reaction buffers, the peak of the identified deiodinated compound was integrated and correlated with the radiation dose (Figure 10). The effect of the radiolysis quencher EtOH was shown to be very effective in the dose ranges up to 117 Gy (equivalent to 36 GBq or 1.8 GBq/mL). In the batch with the highest irradiation dose, the deiodinated compound could be detected in both buffer formulations. Since the primary goal was to identify the major radiolysis product, the irradiation experiment was performed only once (in duplicates).

**Figure 11:**
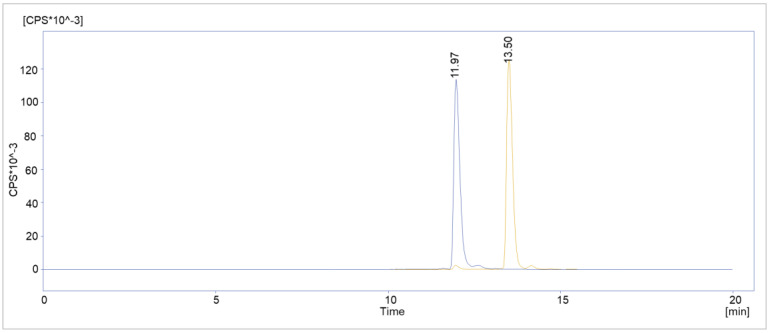
Overlay chromatogram of deiodinated [^177^Lu]Lu-PSMA^I&T^ (blue) and a routine [^177^Lu]Lu-PSMA^I&T^ batch (orange) from the same day.

To further prove the suspicion of deiodination as a radiolysis mechanism, customized “deiodinated” PSMA^I&T^ was labeled with Lutetium-177. Using the optimized synthesis method, the overlay chromatogram showed highly comparable retention times, R_t_ = 11.967 vs. 11.950 min, for the radiolabeled custom peptide and the radiolysis peak of the [^177^Lu]Lu-PSMA^I&T^ routine batch from the same day (Figure 11).

**Figure 12:**
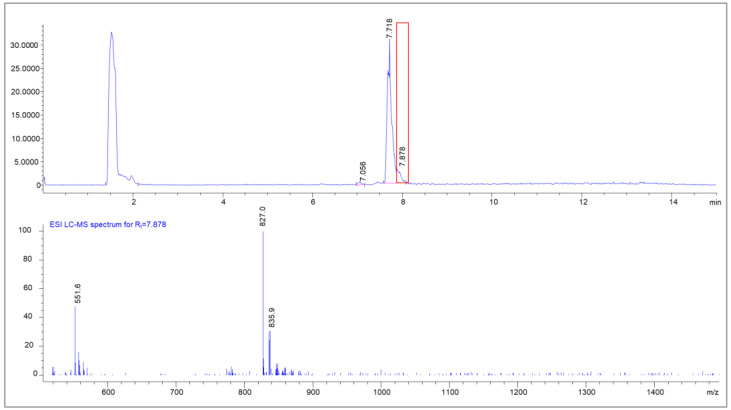
UPLC-MS chromatogram of [^nat^Lu]Lu-PSMA^I&T^ with the MS spectrum of the peak tail (Rt = 7.878 min); clearly visible are condensation products of ^nat^Lu-PSMA (*m*/*z* = 835.0 + [M+2H]^2+^) with the mass of *m*/*z* = 827 [M-H_2_O+2H]^2+^.

Next to the radiolysis side products, a side product in the peak tail of the main product was also found by UPLC-MS. The area showed a mass difference of 18 in comparison to the parent compound, indicating a cyclization process under the dissociation of water (Figure 12).

**Figure 13:**
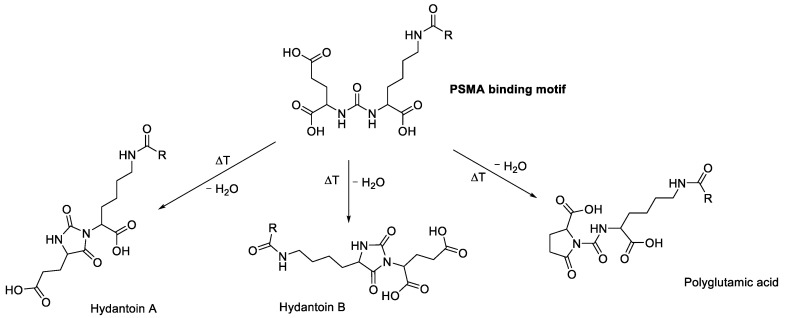
The PSMA binding motif and its possible heat-dependent condensation products, as proposed by Martin et al. [14].

This product was attributed to the heat-dependent condensations of the PSMA-binding motif, as published earlier by Martin et al. [14] for [^177^Lu]Lu-PSMA-617, resulting in three possible side products (Figure 13). As already mentioned in Section 2.2, the condensation products did not correlate with the activity concentration.

### 2.4. Economical Optimization

Lastly, a radiochemical yield analysis of the new production method yielded a possible reduction in starting activity per patient from 9 to 8 GBq. Given the daily limit of 42 GBq Lutetium-177 in our department, serving up to 5 patients per batch became possible due to the successful upscaling process, reducing relative radiation exposure for the involved staff, overall costs and the relative formation of the deiodination product at the same time (Table 2).

## 3. Methods

### 3.1. Chemicals

All chemicals and substances were used as received without further purification. GMP-grade PSMA^I&T^ was purchased from SCINTOMICS (Fürstenfeldbruck, Germany). Custom-synthesized Glu-CO-Lys[(Sub)DLys-DPhe-DTyr-DOTAGA] trifluoroacetate was obtained from piCHEM Forschungs- und Entwicklungs GmbH (Raaba-Grambach, Austria). Sodium ascorbate buffer (Polatom; kit ASC-01 containing 50 mg ascorbic acid and 7.9 mg NaOH) was received from Polatom (Warsaw, Poland) and diluted as indicated with Trace Select™ water (Honeywell Austria, Vienna, Austria). N.c.a. [^177^Lu]LuCl_3_ was purchased either from Isotope Technologies Munich (Munich, Germany) or Isotopia (Petah Tikva, Israel) in GMP quality. For dilutions and HPLC, deionized water generated from a MilliQ device (Merck Millipore) or Aqua ad injectabilia (B. Braun, Maria Enzersdorf, Austria) was used.

### 3.2. Chromatography

#### 3.2.1. UPLC-MS Measurements

Analytical UPLC-MS runs were performed on an Agilent 1260 Infinity II system equipped with a flexible pump, an Agilent 1260 UV detector with variable wavelength (λ = 220 nm) and an LC/MSD mass detector, in combination with either a Chromolith performance RP-18 (100 × 4.6 mm, flow = 1 mL/min) or an Acquity BEH C18 column, 1.7 µm, 3.0 × 50 mm (flow = 0.6 mL/min). A binary mobile phase of H_2_O + 0.1% TFA (A) and acetonitrile + 0.1% TFA (B) was used.

#### 3.2.2. HPLC Measurements

All HPLC measurements of the radioactive products were performed on a VWR Hitachi Chromaster System which included a column oven and UV detection unit, equipped with a Ramona Star Beta radiation detector (Elysia Raytest, Straubenhardt, Germany). HPLC measurements were performed at RT with a UV detection wavelength of 250 nm with a binary mobile phase of H_2_O + 0.1% TFA (A) and acetonitrile + 0.1% TFA (B).

For method 1, a chromolith performance RP-18 (100 × 4.6 mm, flow = 2 mL/min) column (Merck, Darmstadt, Germany) was applied. The respective gradient was 5% to 50% B for 5.5 min.

For method 2, a Jupiter Proteo 4 µm RP (250 × 4.6 mm, flow = 1 mL/min, Phenomenex, Phenomenex Inc., Torrance, CA, USA) was used with the gradient displayed in Table 3. The validation of HPLC method 2 was analyzed via Validat^®^ software (v1) (GUS LAB GmbH, Gera, Germany) and based on the current ICH Q2(R1) and EANM guidelines [17,18].

### 3.3. Validation of HPLC Method 2

Calculations were performed automatically via Validat^®^ software. Intermediate blank injections were performed between measurements and checked for residuals of the respective test compounds. Injection volume was 20 µL for all measurements. Acceptance criteria are displayed in Table 1. Specificity regarding [^177^Lu]Lu-PSMA^I&T^ and the deiodinated product was calculated by Validat^®^ according to the retention times of the substances and the resulting resolution.

For the determination of linearity and LOD/LOQ of the precursor, seven different concentrations between 1 µg/µL and 0.005 µg/µL were prepared by dilution in water for injection. Each concentration was measured as triplicate.

Precision measurement of the reference standard was performed as follows: 10 µL (1 µg/µL) reference standard were mixed with 10 µL water for injection and the measurement was repeated 6 times.

For linearity and LOD/LOQ of the reference standard, seven different concentrations between 1 µg/µL and 0.005 µg/µL were prepared by dilution in water for injection. Each concentration was measured as triplicate. For the determination of the linearity of the radiodetetor, 5 different activities of Lutetium-177 between 3.780 and 0.034 MBq were injected. To determine the repeatability of the radiodetector, a sample of 3.8 MBq was injected 5 times and total peak areas were analyzed.

### 3.4. Radiosyntheses

Radiosyntheses were performed on a Modular-Lab PharmTracer (Eckert & Ziegler GmbH, Berlin, Germany) using the corresponding single-use disposable cassettes.

Before each synthesis, an automated cassette pressure test was performed to ensure the leak-proofness of the synthesis cassette. The production was performed automatically and step-wise by the Modular Lab (version 6.2). The steps are displayed in Table 4.

After each synthesis, a fully automated filter test of the product sterile filter, as programmed in the software, was performed. Differences between the old and new synthesis method are displayed in Table 5.

### 3.5. Syntheses of ^nat^Lu-PSMA^I&T^ and ^nat^Gd-PSMA^I&T^

Briefly, 250 μg (167 nmol) PSMA^I&T^ was dissolved in 200 μL of sodium ascorbate buffer (1 M, pH 4.5). Then, 10 equivalents (1.67 μmol) of either Gadolinium(III) nitrate or Lutetium(III) chloride in 1 mM aqueous solution were added. The vial containing the mixture was heated at 95 °C in an aluminum heating block for 10 min. After cooling to RT, qualitative LC-MS was performed without further workup. LC-MS conditions were RP-LCMS, Chromolith performance; 5–50% MeCN + 0.1% TFA over 10 min.

### 3.6. Dose Calculations

To estimate the radiation dose absorbed in a sample, the contributions of all particles resulting from any decay have to be considered. ^177^Lu decays to ^177^Hf by four possible β^−^ transitions. The relative intensities of these transitions are 11.6%, 0.016%, 8.89% and 79.44% [19]. The average energies <*E_β_*> of the corresponding β particles are 47.23 keV, 78.12 keV, 111.20 keV and 148.84 keV, respectively. During the subsequent transition of the resulting excited state to the ground state additional conversion electrons, Auger electrons, γ photons and X-rays are emitted. The total energy of β particles and electrons is 146 keV (2.34 × 10^−14^ J) per decay and the total energy of photons is 33 keV (5.29 × 10^−15^ J) per decay. If a sample is sufficiently large, the energy of all β^−^ particles and electrons is deposited within the sample. For the dose estimation, the dose of the photons will be neglected, since only a small fraction of the energy will be absorbed within the sample. Thus, the absorbed dose *D* is given by

(1) $$D=\frac{NE_{e^{-}}}{m}$$

where *N* is the number of decays, $E_{e^{-}}$ is the total energy per decay of β particles and electrons and *m* is the mass of the sample.

The number of disintegrations within a time interval *t* is given by

(2) $$N=\int_{0}^{T}Ae^{-\frac{t\ln2}{t_{1/2}}}dt=A\frac{\ln2}{T_{1/2}}\left(1-e^{-\frac{t\ln2}{t_{1/2}}}\right)$$

with *A* being the activity and *t*_½_ the half-life.

If *t* is large with respect to the half-life *t*_½_, *N* approaches $N=A\;\frac{\ln2}{t_{1/2}}$. For *t* much shorter than *t*_½_, *N* can be approximated as N≈A T using the Taylor series of the exponential function. Assuming an approximate density of 1 g/cm^3^ and t much shorter than *t*_½_, the dose can be calculated as

(3) $$D=t{\times A}_{c}\times 2.34\times 10^{-2}Gy*mL$$

where *t* is the time in seconds, *D* is the dose in Gy and *A_c_* is the activity concentration in GBq/mL.

### 3.7. Irradiations

After the preparation of one batch of Gd-PSMA^I&T^ and another batch of Gd-PSMA^I&T^ mixed with 280 μL of ethanol, each batch was divided into 8 portions. The samples were irradiated in duplicates with 4 different doses of X-rays, according to Table 6.

For that, an YXLON reference irradiator (Maxishot, YXLON International GmbH, Hamburg, Germany) was used, as previously described [20]. In brief, irradiation was performed at 200 kV, 20 mA with a focus size of 5.5 mm, using a 0.5 mm copper filter and a 3 mm aluminum filter. The average dose rate was about 1.1 Gy/min. After irradiation, the samples were subjected to LC-MS measurements.

### 3.8. Conclusions

In this study, a thorough analysis of the quality control parameters of [^177^Lu]Lu-PSMA^I&T^ was performed. Major optimizations in HPLC analyses and irradiation experiments followed by UPLC-MS studies revealed a significant contribution of cyclization and radiolysis products to the amount of radioactive impurities in the production of [^177^Lu]Lu-PSMA^I&T^.

The pre-peak, now known to consist of deiodinated [^177^Lu]Lu-PSMA^I&T^ and induced by radiolysis, is expected to show altered pharmacokinetics and might reduce the overall tumor dose, but still demonstrated cell uptake in a previous study [15]. Our findings regarding the identity of the pre-peak are in accordance with a recently published study [11] and corroborate them by the approach of radiolabeling of the suspected radiolysis product and comparing the HPLC retention time with that of a [^177^Lu]Lu-PSMA^I&T^ routine batch.

The cyclization products were already shown to lack tumor cell binding, due to the altered PSMA binding motif. Further, NMR studies were performed to elucidate the definite structure of the cyclization products [14]. In our study, the conclusion was drawn based on the combination of UPLC-MS, HPLC elution profile and independency from activity concentration.

A risk-based approach resulted in the extension of our radiochemical purity thresholds for the sum of hydantoins and deiodinated product for 90%, given the fact that the cyclization products constitute the most critical impurity with respect to therapeutic efficacy and only represented 2.87 ± 0.85% of the total radioactivity in the samples. As expected, the formation of the main radiolysis product was shown to depend on the activity concentration and represented 2.8 ± 0.1% of the total radioactivity in the samples.

To our knowledge, this is the first time that the discussed cyclization peaks were shown to contribute to the overall amount of radiochemical impurities in [^177^Lu]Lu-PSMA^I&T^ and the question of how adequate HPLC methods should be used to detect them was addressed.

Further, an overall improvement of processes for the routine supply of [^177^Lu]Lu-PSMA^I&T^ was achieved and it was possible to keep up with the increasing demand for this radioligand therapy. Due to the reduction in starting activity, a possible cost reduction through quality assurance procedures was also shown in this work.

## Figures and Tables

**Table 1:** Validation of HPLC method 2.

Radiodetector				
Parameter	Radiochemical Identity	Radiochemical Purity	Acceptance Criteria	Results
Precision (repeatability)	CV% ≤ 5%		complies	
Specificity (radiolysis product)	Rs ≥ 1.5		complies	
Linearity	R2 ≥ 0.99		complies	
**UV detector**				
**Parameter**	**Acceptance Criteria**		**Results**	
Precision (repeatability)	CV% ≤ 5%		complies	
LOD ([^nat^Lu]Lu-PSMA^I&T^)	Based on calibration curve		0.0361 µg/µL	
LOD (PSMA^I&T^)	Based on calibration curve		0.0149 µg/µL	
LOQ ([^nat^Lu]Lu-PSMA^I&T^)	Based on calibration curve		0.1269 µg/µL	
LOQ (PSMA^I&T^)	Based on calibration curve		0.0531 µg/µL	
Linearity [^nat^Lu]Lu-PSMA^I&T^	R2 ≥ 0.99		complies	
Linearity PSMA ^I&T^	R2 ≥ 0.99		complies	

**Table 2:** Retrospective analysis of 26 routine batches of [^177^Lu]Lu-PSMA^I&T^ with average yields shows excellent yields with the possibility of reduction in starting activity; SA = starting activity; * 1 patient receives a therapeutic dose of 7.4 GBq.

Patients */Batch	SA Original Method [GBq]	SA Adapted Method [GBq]	av. RCY [%]	av. Absolute Yield [GBq]	Minimum Final Activity [GBq]
5	40	-	97.7 ± 0.5	41.5 ± 0.2	37.0
4	36	32	96.3 ± 1.2	36.8 ± 0.8	29.6
3	27	24	96.5 ± 2.6	27.4 ± 2.0	22.2
2	18	16	93.6 ± 5.3	17.6 ± 1.3	14.8

**Table 3:** Solvent gradient for HPLC method 2 (flow = 1 mL/min).

Time [min]	A (%)	B (%)
1	90	10
2	88	12
3	84	16
5	80	20
7	76	24
8	74	26
9	72	28
10	71	29
11	70.5	29.5
12	70	30
14	69.5	30.5
17	5	95
18	95	5
20	95	5

**Table 4:** Automated radiosynthesis procedure; the reaction buffer, radiosynthesis conditions in step 3 and the final formulation buffer and volume in step 6 vary between the original and adapted method and are described in Table 5.

#	Step
1	Conditioning of the Sep-Pak^®^ C18 Plus cartridge with water/ethanol (50/50 mixture) followed by the formulation buffer
2	Transfer of radioactivity to reactor and rinsing of the activity vial with 1.4 mL of reaction buffer
3	Radiosynthesis
4	Transfer of the reaction mixture to the Sep-Pak^®^ C18 Plus Cartridge
5	Elution of the product with EtOH/H_2_O (2.5 mL, 50/50 mixture)
6	Formulation of the product to a final volume of 20 mL with formulation buffer

**Table 5:** Differences between the original and the adapted synthetic procedure.

Parameter	Original Method	Adapted Method
Product vial preparation	Sterile filtration of 0.15 mL (=30 mg DTPA) Ditripentat-Heyl^®^ (200 mg/mL) solution into product vial	Sterile filtration of 0.15 mL (=30 mg DTPA) Ditripentat-Heyl^®^ (200 mg/mL) solution and 1.4 mL reaction buffer into product vial
Reaction buffer	35.7 mg/mL L (+)-ascorbic acid, 11.1 mg/mL NaOH (commercial buffer kit, Polatom^®^) in 1.4 mL Trace Select^®^ water	92.1 mg/mL L (+)-ascorbic acid, 111.4 mg/mL sodium acetate trihydrate, 34 mg/mL gentisic acid in 1.4 mL water for injection, adjusted to pH 5.2 with 2 N NaOH
Precursor amount	125 µg/~9 GBq n.c.a. Lutetium-177	
EtOH present during synthesis	200 µL+ 0.5 µL per µg precursor	0.5 µL per µg precursor
T [°C]	90	95
t [min] labelling reaction	10	30
Formulation buffer	16 mL phys NaCl 0.9%	24 mg/mL sodium ascorbate + 2.4 mg/mL L (+)-ascorbic acid in 16 mL phys NaCl 0.9%
Total volume EOS [mL]	18.6	20.0

**Table 6:** Irradiation parameters (actual dose and calculated activity equivalents) of the irradiated samples.

Sample Number Gd-PSMA^I&T^	Sample Number Gd-PSMA^I&T^ + EtOH	Dose [Gy]	Activity Equivalent for 45 min Storage Time [GBq]
1.1	2.1	22.8	7
1.2	2.2	58.6	18
1.3	2.3	117.1	36
1.4	2.4	130	40

## Data Availability

Data can be provided on an individual basis by the authors.
